# 3D facial mask for facial asymmetry diagnosis

**DOI:** 10.1016/j.heliyon.2024.e26734

**Published:** 2024-02-23

**Authors:** Liang Lyu, Ming-Jin Zhang, Ao-Nan Wen, Shuo Wang, Yi-Jiao Zhao, Yong wang, Ting-Ting Yu, Dawei Liu

**Affiliations:** a Department of Orthodontics, Peking University School and Hospital of Stomatology & National Center of Stomatology, Beijing, China; b National Clinical Research Center for Oral Diseases & National Engineering Research Center of Oral Biomaterials and Digital Medical Devices, Beijing, China; c Beijing Key Laboratory of Digital Stomatology, Beijing, China; d Center of Digital Dentistry/Department of Prosthodontics, Peking University School and Hospital of Stomatology, Beijing, China; e National Center of Stomatology, Beijing, China; f Research Center of Engineering and Technology for Computerized Dentistry Ministry of Health, Beijing, China

**Keywords:** Facial asymmetry, Anthropometric analysis, Three-dimensional, Esthetic evaluation

## Abstract

**Objectives:**

Facial asymmetry is a common problem seen in orthodontic clinics that may affect patient esthetics. In some instances, severe asymmetry that affects patient esthetics may cause psychological issues. An objective method is therefore required to help orthodontists identify asymmetry issues.

**Materials and methods:**

We used three-dimensional (3D) facial images and landmark-based anthropometric analysis to construct a 3D facial mask to evaluate asymmetry. The landmark coordinates were transformed using a symmetric 3D face model to evaluate the efficacy of this method. Patients with facial asymmetry were recruited to conduct mirror and overlap analysis to form color maps, which were used to verify the utility of the novel soft tissue landmark-based method.

**Results:**

The preliminary results demonstrated that the asymmetry evaluation method had a similar response rate compared to diagnosis using mirror and overlap 3D images, and could therefore identify 3D asymmetry problems.

**Conclusions:**

By using 3D facial scans and 3D anthropometric analysis, we developed a preliminary evaluation method that provides objective parameters to clinically evaluate patient facial asymmetry and aid in the diagnosis of asymmetric areas.

**Clinical relevance:**

This study presents a novel facial asymmetry diagnostic method that has the potential to aid clinical decisions during problem identification, treatment planning, and efficacy evaluation.

## 1 Introduction

Facial asymmetry describes two halves of a face that are not perfectly matched in terms of size and shape [1]. Facial symmetry is considered an essential factor for desirable esthetics, which is vital in orthodontics and orthognathics [2,3].

The current common diagnosis of facial asymmetry involves clinical evaluation, photography, and radiography, which may sometimes cause bias in individual judgment [[4], [5], [6]]. Radiological examination has traditionally been the gold standard for facial asymmetry evaluation [7,8]. While facial symmetry may be impacted by skeletal tissue, soft tissue forms the facial appearance and ultimately determines facial symmetry [[9], [10], [11], [12], [13]].

For two-dimensional (2D) images, posture, capture angle, and light during image acquisition affects the images and judgment of symmetry, resulting in bias. In addition, 2D-based diagnoses may lack diagnostic dimension [14]. Thus, three-dimension (3D) facial analysis at the soft tissue level may be a straightforward method for evaluating facial asymmetry.

With the advent of 3D imaging and anthropometric analysis technology, new anthropometry based on 3D imaging is on a tear. The current evaluation methods of asymmetry based on 3D facial images includes expert judgment, facial asymmetry index (FAI) measures, and statistical shape analysis [7,[15], [16], [17]]. Wu et al. recruited senior doctors to compare the evaluation of patients with asymmetry using multiple methods > [15]. Huang et al. measured the FAI values in 60 healthy adults to construct clinical asymmetry evaluation criteria for Chinese people [16]. Although such methods are considered standard in the diagnosis of facial asymmetry, judgment bias and mathematical complexity indicate that a more objective and simple method is required.

Some studies have used the abstracted facial linear model to describe facial deformity [18,19]. However, this linear model is based on the average facial information of a specific race and therefore may have some weaknesses in generalizability. In addition, such a model has a rough perceptual cognition for general facial deformities.

In this study, we developed a landmark-based facial asymmetry evaluation method to quantitatively describe the location and severity of facial asymmetry.

## 2 Materials and Methods

### 2.1 Construction of the 3D facial mask

By combining previous studies on soft tissue landmarks for facial deformity evaluation [[20], [21], [22], [23], [24]], 34 soft tissue markers were chosen to form the 3D facial mask, and the coordinates of facial anatomical landmarks on patient images were determined using Geomagic Studio 2013 software (3D Systems, Rock Hill, SC).

After locating the landmarks, the points were connected to form angle parameters; thus, a wireframe mask was constructed to illustrate asymmetry at the 3D soft tissue level.

**Fig. 1:**
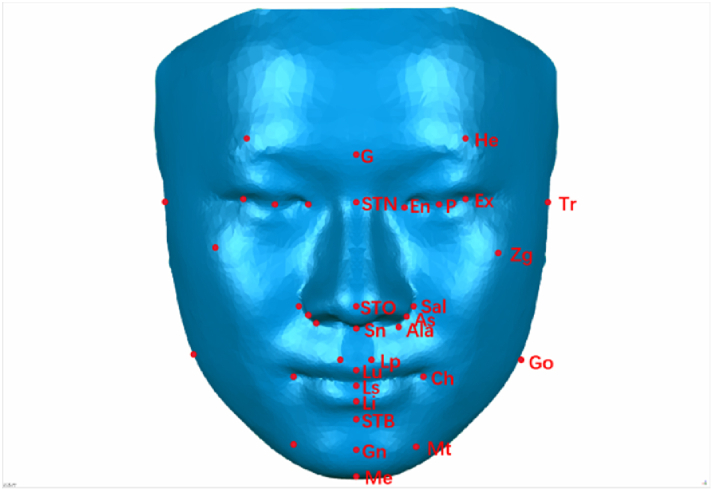
Landmarks of the wireframe 3D facial mask.

**Fig. 2:**
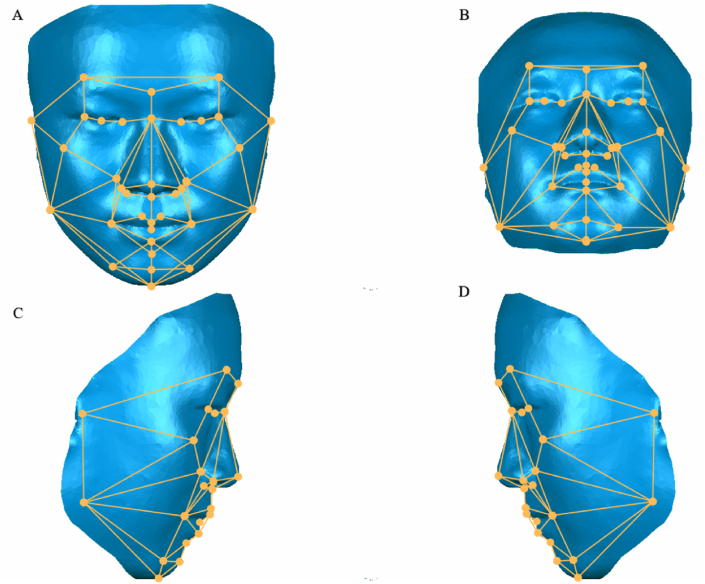
Wireframe 3D facial mask.

The 3D facial average model used in a previous study (Fig. 1) [25] was built to form a symmetrical 3D facial mask. Frontal, lateral, and upward views of the 3D facial mask are shown in Fig. 2A–D. The names, abbreviations, and definitions of the landmarks are listed in Table S1.

**Fig. 3:**
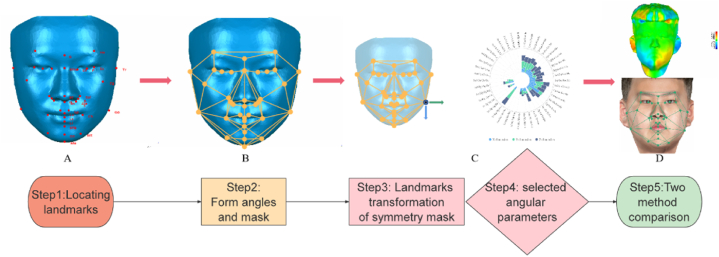
Flowchart.

After constructing the 3D facial mask, further tests were conducted to examine the mechanism and efficiency of the proposed method. A flowchart summarizing the study design is shown in Fig. 3.

### 2.2 Facial data collection

Facial data of the patients were collected to test the utility of the 3D facial mask. All 3D facial images were obtained using the Bellus 3D Arc 1 system (Bellus3D, Inc. Campbell, CA, USA). The participants were recruited from patients attending the Department of Orthodontics, University Stomatology Hospital. The inclusion criteria were as follows: identifiable ethnic origin, age between 8 and 15 years, presence of malocclusion, average body mass index, and absence of inherited craniofacial deformities. Ethical approval for this study and informed consent were obtained from the relevant institutional review boards and the study participants.

The Arc 1 camera was fixed at a standardized static position with a central point. The poses of the patients were standardized by placing them at the center of the camera aperture in the occlusal rest position

### 2.3 Regions of interest selection and landmark coordinate transformation

**Fig. 4:**
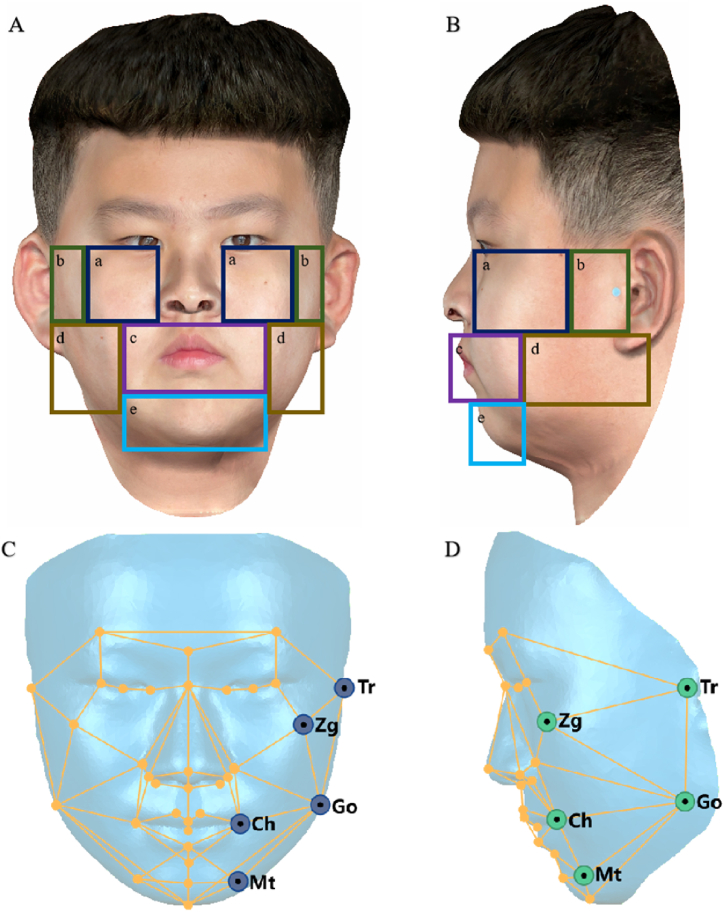
The five main regions and landmarks considered in this study.

To examine the efficacy of the 3D facial mask, we divided the 3D data surface of the face into five areas (labia, mandible angular, cheek, chin, and articularis), as shown in Fig. 4A and B. Five major landmarks (Go, Ch, Mt, Tr, and Zg) were selected in the facial mask to represent the five main areas illustrated in Fig. 4C and D:

The Go, Ch, Mt, Tr, and Zg coordinates were artificially transformed separately using a symmetrical 3D facial mask to simulate facial asymmetry in different areas. The left landmarks were chosen for the transformation based on a previous study [5]. Each point was changed 5 mm in three dimensions: the X-axis for transverse dimension transformation, the Y-axis for vertical dimension transformation, and the Z-axis for sagittal dimension transformation [26,27]. After changing the landmark coordinates, subsequent changes in angular parameters were recorded and clinical parameters were selected for asymmetry evaluation. Changes in angle parameters were documented using a polar chart.

### 2.4 Comparisons of mirror-overlap analysis and the 3D facial mask

Based on a previous study [13], we developed an angular asymmetry index formula for angle differences to avoid bias.

The asymmetry indexes were calculated with the following formula:

(1) $$\begin{array}{c} A\text{ngle}\;\text{asymmetry}\;\text{index}＝\frac{\left|R-L\right|}{L}\times 100\% \end{array}$$

where R is the angular parameter of the right side of the face, and L is the angular parameter of the left side. In this study, an angular asymmetry index of >2% was considered a positive difference [28].

The 3D surface image of the original face was mirrored using Geomagic. The original and mirror images were then registered with the forehead and upper nasal region [29], a best-fit alignment was used, and the stack images were subjected to deviation analysis using the bias analysis function of the software. Based on previous studies [30], we set the threshold level as ±2 mm: a bias in a location greater than 2 mm was considered to occur asymmetrically. A color map was used to demonstrate the deviations of the mirror and overlap 3D facial image [31].

Comparisons of the two methods were made in the five areas shown in Fig. 4, where areas with deviation parameters greater than 2 mm after mirrored overlap were used as positive areas. Positive regions were recorded, and whether their corresponding 3D facial masks could detect this positive bias was documented. The number of responses at each site was summed, the total positive regions from the number of mirror and overlap analysis were noted as M, and the number of 3D facial mask calls was denoted as T.

The response rate was calculated with the following formula:

(2) $$\begin{array}{c} \text{Response}\;\text{rate}＝T/M\times 100\% \end{array}$$

### 2.5 Statistical analysis

All statistical analyses were performed using the Statistical Package for Social Sciences software (version 25.0; SPSS Inc., Chicago, Illinois, USA). Descriptive statistics were used to evaluate the consistency of the mirror and overlap results and 3D facial wireframe mask diagnosis results.

## 3 Results

### 3.1 Mechanism of parameters in the 3D facial mask

**Fig. 5:**
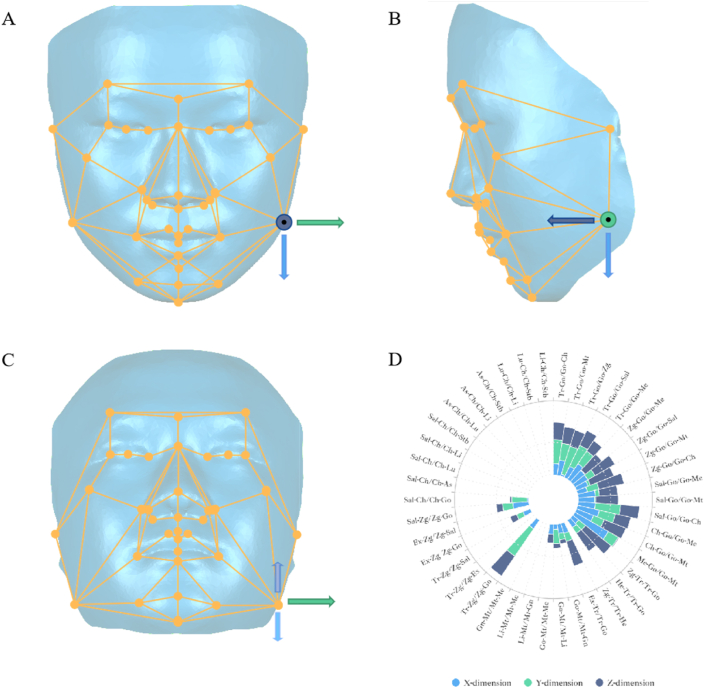
Artificial transformation of Go's 3D coordinates in a wireframe 3D facial mask.

Five areas of interest (labia, mandible angular, cheek, chin, and articularis) and the artificial transformation of coordinates were assessed in the symmetrical 3D facial mask (Fig. 5A–C). From this series of transformations, we found that the evaluation method parameters responded to asymmetric changes in a 3D direction. We used Go as a representation of mandibular angle asymmetry and found 25 positive angular parameters (Fig. 5D). The most sensitive parameters were Me-Go/Go-Mt for the X (transverse) dimension, Tr-Zg/Zg-Go for the Y (vertical) dimension, and Zg-Tr/Tr-Go for the Z (sagittal) dimension. Taking the Ch point as a representation of the asymmetry of the labial region, we found that 16 angular parameters were positive and covered three dimensions: Gn-Mt/Mt-Me for the X (transverse) dimension, Li–Mt/Mt-Gn for the Y (vertical) dimension, and Me-Go/Go-Mt for the Z (sagittal) dimension (Fig. S1D). Taking Zg as a representation of the asymmetry of the cheek region, 13 positive angular parameters were found, and the most significant parameters were Ex-Zg/Zg-Sal for the X (transverse) dimension, Zg-Tr/Tr-He for the Y (vertical) dimension, and Tr-Zg/Zg-Ex for the Z (sagittal) dimension (Fig. S2D). Taking Mt as a representation of chin asymmetry, we found that 11 angular parameters were positive, and the most sensitive parameters were Gn-Mt/Mt-Me for the X (transverse) dimension, Li–Mt/Mt-Gn for the Y (vertical) dimension, and Me-Go/Go-Mt for the Z (sagittal) dimension (Fig. S3D). Tr was regarded as one of the most important landmarks for ramus and joint region asymmetry, and after artificial coordinate transformation, we found 12 positive angular parameters, and the most sensitive were Ex-Tr/Tr-Go for the X (transverse) dimension, Tr-Zg/Zg-Go for the Y (vertical) dimension, and Tr-Go/Go-Zg for the Z (sagittal) dimension (Fig. S4D).

### 3.2 Practicable clinical parameters in the 3D facial mask

Thirteen spatial angles were used to evaluate 3D asymmetry in the five major regions. An overall illustration of these parameters and their relationship with each dimension of each region is shown in Table 1 and Fig. S5.

**Table 1:** Evaluations of the wireframe 3D facial mask.

Position of asymmetry	Dimension of asymmetry	Angular parameters
Labia	X	Sal-Ch/Ch-Stb
	Y	Sal-Go/Go-Ch
	Z	Sal-Ch/Ch-As
Mandible angular	X	Me-Go/Go-Mt
	Y	Tr-Zg/Zg-Go
	Z	Zg-Tr/Tr-Go
Cheek	X	Ex-Zg/Zg-Sal
	Y	Zg-Tr/Tr-He
	Z	Tr-Zg/Zg-Ex
Chin	X	Gn-Mt/Mt-Me
	Y	Li–Mt/Mt-Gn
	Z	Me-Go/Go-Mt
Articularis	X	Ex-Tr/Tr-Go
	Y	Tr-Zg/Zg-Go
	Z	Tr-Go/Go-Zg

### 3.3 Parameters of the labia region

Sal-Ch/Ch-Stb(∠Sal-Ch-Stb) represents the asymmetry level in the X (transverse) dimension. Sal-Go/Go-Ch(∠Sal-Go-Ch) represents the asymmetry level in the Y (vertical) dimension. Sal-Ch/Ch-As(∠Sal-Ch-As) represents the asymmetry level in the Z (sagittal) dimension.

### 3.4 Parameters of the mandible angular region

Me-Go/Go-Mt(∠Me-Go-Mt) represents the asymmetry level in the X (transverse) dimension. Tr-Zg/Zg-Go(∠Tr-Zg-Go) represents the asymmetry level in the Y (vertical) dimension. Zg-Tr/Tr-Go(∠Zg-Tr-Go) represents the asymmetry level in the Z (sagittal) dimension.

### 3.5 Parameters of the cheek region

Ex-Zg/Zg-Sal(∠Ex-Zg-Sal) represents the asymmetry level in the X (transverse) dimension. Zg-Tr/Tr-He(∠Zg-Tr-He) represents the asymmetry level in the Y (vertical) dimension. Tr-Zg/Zg-Ex(∠Tr-Zg-Ex) represents the asymmetry level in the Z (sagittal) dimension.

### 3.6 Parameters of the chin region

Gn-Mt/Mt-Me(∠Gn-Mt-Me) represents the asymmetry level in the X (transverse) dimension. Li–Mt/Mt-Gn(∠Li-Mt-Gn) represents the asymmetry level in the Y (vertical) dimension. Me-Go/Go-Mt(∠Me-Go-Mt) represents the asymmetry level in the Z (sagittal) dimension.

### 3.7 Parameters of the articularis region

Ex-Tr/Tr-Go(∠Ex-Tr-Go) represents the asymmetry level in the X (transverse) dimension. Tr-Zg/Zg-Go(∠Tr-Zg-Go) represents the asymmetry level in the Y (vertical) dimension. Tr-Go/Go-Zg(∠Tr-Go-Zg) represents the asymmetry level in the Z (sagittal) dimension.

It should be noted that Me-Go/Go-Mt can reflect the X (transverse) dimension asymmetry in the mandible angular region or the Z (sagittal) dimension asymmetry in the chin region. Furthermore, Tr-Zg/Zg-Go can reflect the Y (vertical) dimension asymmetry in the mandible angular region or Y (vertical) dimension asymmetry in the articularis region. In this case, we should combine the parameters of each region and perform a comprehensive analysis.

### 3.8 Application and preliminary inspection of the 3D facial mask

**Fig. 6:**
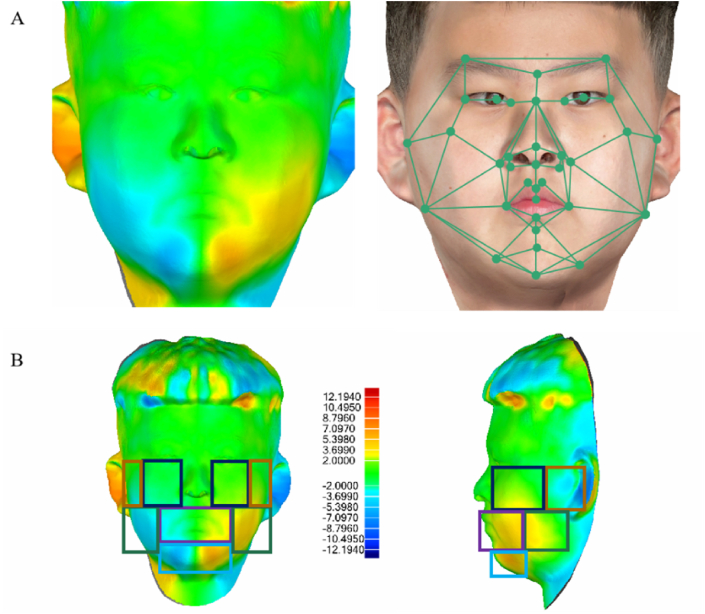
The two diagnostic methods for facial asymmetry. The color map indicates the magnitude of the asymmetry at each point on the full face: yellow to red indicates asymmetry larger than 2 mm, light blue to dark blue indicates asymmetry larger than -2mm, and green indicates no asymmetry.

To further illustrate this method, subjects were evaluated using a 3D facial mask, which was mirrored and superimposed on a 3D facial scan. A color map was generated to show the variations in mirrored and overlapped 3D faces [9,[32], [33], [34]]. Based on previous studies [9,35], the mirror and overlap difference thresholds in this study were set to 2 mm. The process demonstration is shown in Fig. 6A and B, and the detailed diagnostic information extracted from the 3D facial mask is shown in Table 2.

**Table 2:** Outcomes extracted from the 3D facial mask.

Region	Dimension	Parameters	Differences
Labia	X	Sal-Ch/Ch-Stb	5%
Labia	Y	Sal-Go/Go-Ch	2%
Labia	Z	Sal-Ch/Ch-As	1%
Mandible angular	X	Me-Go/Go-Mt	20%
Mandible angular	Y	Tr-Zg/Zg-Go	2%
Mandible angular	Z	Zg-Tr/Tr-Go	5%
Cheek	X	Ex-Zg/Zg-Sal	2%
Cheek	Y	Zg-Tr/Tr-He	2%
Cheek	Z	Tr-Zg/Zg-Ex	2%
Chin	X	Gn-Mt/Mt-Me	7%
Chin	Y	Li–Mt/Mt-Gn	8%
Chin	Z	Me-Go/Go-Mt	20%
Articularis	X	Ex-Tr/Tr-Go	3%
Articularis	Y	Tr-Zg/Zg-Go	2%
Articularis	Z	Tr-Go/Go-Zg	4%

Twenty-five patients with facial asymmetry were recruited, and mirror and overlap analyses and 3D facial mask evaluations were conducted. The response rates are shown in Table 3. The 3D facial mask had an overall response rate of 94.1% for facial asymmetry among the 25 patients. The labia region had 100% of the response rate (Spearman's ρ = 0.457), and the mandible angular region had 95.5% of the response rate (ρ = 0.510). The response rate of the chin, cheek, and articularis region was 87.0% (ρ = 0.600), 91.7% (ρ = 0.482), and 95.2% (ρ = 0.873), respectively. A two-sample *t*-test was used to compare the identified cases by mirror recognition and 3D facial mask recognition, and there was no significant difference in the recognition results of facial asymmetry between the gold standard method and 3D facial mask analysis method (P = 0.6938).

**Table 3:** Examination response rate.

Region	Mirror and overlap	3D facial mask	Response rate
Labia	23	23	100.0%
Mandible angular	22	21	95.5%
Cheek	12	11	91.7%
Chin	23	20	87.0%
Articularis	21	20	95.2%
Overall	101	95	94.1%

## 4 Discussion

Facial asymmetry involves 3D factors that can affect one's outlook on their esthetics, which can impact their mental health. Issues surrounding asymmetry occur in different dimensions and locations, often leading to diverse diagnoses and treatment methods; therefore, it is important to recognize 3D problems for clinical evaluation in orthodontics and orthognathics. With the development of digital technology, facial asymmetry diagnosis and treatment in orthodontics and orthognathic surgery combined with static or dynamic 3D facial data has become a growing field.

Current studies on the digital diagnosis of facial asymmetry often focus on building an average face for a specific ethnic group [19] or obtaining a 3D facial symmetry reference plane (SRP) [36,37]. Because perfect facial symmetry is difficult to obtain [38,39], it may be challenging for orthodontists to reach a consensus on identifying the SRP. A single reference plane may improve the diagnosis of the 3D problem due to the asymmetry of the three dimensions. There may be a need for further construction of standard reference planes in 3D simultaneously, which increases the difficulty of evaluation.

The treatment of facial asymmetry often includes orthodontics and orthognathic surgery [40]. Autologous fat transfer, dermal grafts, local flaps, and free flap transplantation can help rebuild the symmetry of the face contours [[41], [42], [43]]. However, despite effective treatment, the diagnostic analysis or effect evaluation of most case reports was performed using posteroanterior radiography and 2D photography, which focus on the midline, skeletal landmarks, or personal judgment [[44], [45], [46]]. Our 3D facial mask may provide a simple way to help clinicians more conveniently and visibly evaluate not only the problem of asymmetry, but also therapeutic effectiveness.

The use of facial soft tissue landmarks to evaluate facial asymmetry or other facial dysmorphisms has been reported in many studies [[20], [21], [22], [23], [24]]. These studies have confirmed the reliability of soft tissue landmarks and their essential role in evaluating facial morphology. Therefore, we developed a landmark-based analysis method for 3D facial images to quantify the occurrence of asymmetry without requiring a specific reference plane. A patient's facial information can be abstracted as a linear-formed wireframe 3D facial mask, and the facial-angle information of the mask frame can be obtained. Such information can help identify the degree, exact location, and dimensions of facial asymmetry.

By identifying facial landmarks, the spatial angular parameters can be calculated and used to diagnose facial asymmetries. In this study, we selected 13 angle parameters to illustrate which dimension and facial region asymmetries occur. In addition, 3D facial masks constructed using angle parameters can provide clinicians with a visual illustration of asymmetry problems. During clinical practice, changes in angular parameters can aid orthodontists in making clinical judgments and evaluating curative effects.

Mandibular asymmetry related to functional adaptation has also been observed. Through cone beam computed tomography analysis, research has shown that functional posterior crossbite may cause adaptive asymmetric remodeling of the mandible [47]. Orthodontists are increasingly evaluating the curative effect of orthodontic treatment, such as maxillary expansion in functional asymmetry caused by unilateral posterior crossbite [48]. The extent to which functional malocclusion and orthodontic treatment methods affect soft tissue facial symmetry should be further explored quantitatively using face scanning and quantitative detection methods.

Different landmarks in 3D facial masks can result in various recognition errors. A previous study found that midline landmarks had a smaller error range than bilateral landmarks [49]. This may lead to bias when constructing 3D facial masks, and we are in the process of conducting further studies to reinforce accuracy and avoid bias.

The frame mask can illustrate the asymmetry condition qualitatively; however, there are still problems in determining the mechanism of the asymmetry occurrence dimension and location in this mask, as well as how to quantitatively describe the severity of asymmetry in a simple and convenient manner. To further explore the mechanism of this method and examine its efficacy, a symmetrical face model was used to form a symmetrical 3D facial mask, and then some landmark coordinates were artificially changed to simulate morphological changes in patients. A previous study proposes that observers more easily perceive left face asymmetry than right [5], which may be caused by “hemispheric laterality” [[50], [51], [52]]. McAvinchey et al. found that 5 mm is the threshold for nonprofessionals to recognize asymmetry in 3D face data [26]. Hence, a 5 mm coordinate transformation of the five selected facial areas was conducted to simulate the asymmetry change. Based on the results, the mask system was simplified, and 13 angles were selected to effectively show orthodontists where, and more importantly, which dimension of asymmetry occurred.

Currently, the angle parameters for a 3D face still lack a standard between the left and right side of faces. Some believe that angular parameter deviations of less than 2–4° are not generally considered asymmetric [35]. A similar study used the facial asymmetry index formula to calculate line distance differences [13], from which we developed the angular asymmetry index formula for angle differences in this study so as to avoid bias. We set deviations of more than 2% as the symmetry threshold. Future studies on the reference range of 3D plane angle measurements are being conducted to reinforce the credibility of this 3D facial mask.

To further verify the utility of this method, patients with facial asymmetry were recruited, and a 3D facial mask evaluation and mirror and overlap analysis were conducted. Mirror and overlap analysis is a standard method for assessing facial asymmetry by superimposing an original 3D facial image on its mirror copy [53]. This method has been previously described and is regarded as a relatively reliable diagnostic method [9,[32], [33], [34]]. Lum et al. classified the asymmetry of mirror and overlap data as mild (less than 2 mm), moderate (2–5 mm), or severe (>5 mm) [9]. Another study indicated that the human eye cannot detect facial asymmetry less than 2 mm, but can confidently identify asymmetry greater than 5 mm [35]. Hence, we used 2 mm as the asymmetry threshold for the mirror and overlap images and tested the response rate of each of the five selected mean regions of interest. We found that the 3D facial mask had a 94.1% overall response rate for facial asymmetry among all patients in this study. The labia and mandible angular regions had 100% and 95.5% response rates, respectively, indicating the high relative accuracy of these areas. In addition, the response rates in the chin and cheek regions were 87.0% and 91.7%, respectively, indicating that further efforts are required to improve this method.

Using the mirror and overlap method as a standard, we found good agreement between the two methods. This verified that the 3D facial mask was reliable for evaluating clinical asymmetry. In addition, compared to the mirror and overlap method, the 3D facial mask could further distinguish the specific dimensions where asymmetry occurs and develops, and may therefore aid clinicians in patient diagnosis and therapeutic decisions.

Although mirror and overlap methods are relatively simple, the manual marking of landmarks can be time consuming and prone to errors. Similarly, although artificial intelligence (AI)-based landmark recognition may be more effective, errors or biases in algorithms may introduce significant systematic errors that may be greater than the actual patient asymmetry. However, a recent AI study for marking facial landmarks demonstrated progress, with an average error of 1.65 ± 1.19 mm [25]. AI has the potential for feature extraction and can help clinicians automatically detect facial landmarks. There has been increased interest in research related to this AI feature detection field, including facial asymmetry evaluation [8,[54], [55], [56]]. With the application of this technology, traditional landmark-based analysis may have the advantages of not only visualization, but also reliability and simplicity.

For patients with severe asymmetric and dysmorphic faces, a clear problem recognition method is required to help clinicians determine the specific location and direction of facial asymmetry, and to use objective data to quantify the problem more accurately. A 3D facial mask can generate objective line-angle data, similar to cephalometric data, which numerically demonstrates facial asymmetry. After the construction of a 3D facial mask, line graphics can simplify complex facial information and better assist in judgment. In addition, diagnostic data can distinguish between asymmetry problems in different dimensions, thereby helping practitioners identify the specific direction and dimensions of asymmetry. In future studies, further exploration will be conducted with increased sample sizes and a greater number of patients with severely asymmetric and dysmorphic faces to explore the utility of 3D facial masks for clinical diagnosis and treatment.

A limitation of this study is that there is still a need to optimize the angle measurement indicators to identify and diagnose problems. Follow-up studies require increased sample size and diversity to improve the application value of 3D facial masks. It is worth noting that soft tissue, such as skin, may have age-related changes, and increases in wrinkles and skin relaxation may affect and change the diagnosis and evaluation of facial asymmetry. This study has not yet covered the bias caused by age-related changes, and therefore we will further expand the sample size to include a wider range of ages to better explore the diagnosis and evaluation of lower asymmetry. Moreover, because the information extracted by the 3D facial mask varies, it can be applied to more than just asymmetric evaluations. Therefore, the application scope of anthropologic analysis for esthetic assessment at the soft tissue level requires future research.

## 5 Conclusion

Based on landmarks for soft tissue anthropometric analysis, we propose a quantitative method that uses an abstract 3D facial template mask to analyze 3D facial asymmetry. The 3D facial mask exhibited certain advantages and reliability in preliminary testing. This method can provide new insights for clinical diagnosis, treatment plan design, and efficacy evaluation.

## Funding

Publication charges, articles purchases and information gathered for this article was mutually supported by the 10.13039/501100001809National Natural Science Foundation of China (No. 81970909, 82271009) to DL; Young Scientists Fund of PKUSS (No. 20110203, No. 20170109) to DL; the 10.13039/501100007937Peking University Medicine Seed Fund for Interdisciplinary Research (BMU2018MX007) to DL; the Fund of the 10.13039/501100011262State Key Laboratory of Oral Disease, 10.13039/501100004912Sichuan University (SKLOD2021OF09); Key R & D Plan of Ningxia Hui Autonomous Region No. 2020BCG01001(D.L.); China oral disease foundation(Grant/Award Number: A2021-057); National multidisciplinary cooperative diagnosis and treatment capacity building project of PKUSS(PKUSSNMP-202020); the New Clinical Technology Fund of PKUSS [PKUSSNCT-20A07] to DL.

## Ethics approval and consent to participate

Ethical approval was obtained from the Ethics Committee of the University School and Hospital of Stomatology (NO.PKUSSIRB2021026208). Informed consent was obtained from all patients.

## Consent to publish

Not applicable.

## Data availability statement

The datasets generated and/or analyzed during the current study are not publicly available because of concerns that publication of information might impact participants’ anonymity, but are available from the corresponding author upon reasonable request.

## CRediT authorship contribution statement

**Liang Lyu:** Writing – review & editing, Writing – original draft, Visualization, Validation, Software, Project administration, Methodology, Investigation, Formal analysis, Data curation, Conceptualization. **Ming-Jin Zhang:** Writing – review & editing, Writing – original draft, Validation, Software, Methodology, Investigation, Formal analysis, Data curation, Conceptualization. **Ao-Nan Wen:** Software, Investigation, Data curation. **Shuo Wang:** Writing – original draft, Supervision. **Yi-Jiao Zhao:** Supervision, Resources, Methodology, Conceptualization. **Yong wang:** Writing – original draft, Supervision, Conceptualization. **Ting-Ting Yu:** Writing – original draft, Conceptualization. **Dawei Liu:** Writing – review & editing, Writing – original draft, Visualization, Validation, Supervision, Software, Resources, Project administration, Methodology, Investigation, Funding acquisition, Formal analysis, Data curation, Conceptualization.

## Declaration of competing interest

The authors declare no conflict of interest.
