# Supplementary material for: 3D facial mask for facial asymmetry diagnosis
Source: Heliyon. 2024 Feb 23;10(5):e26734. doi: 10.1016/j.heliyon.2024.e26734 (PMC10912245; doi:10.1016/j.heliyon.2024.e26734)

**Supplemental Table**

Table S1. The landmarks used in this system.

| Landmark | Abbreviation | Definition | Midline or bilateral landmark |
| --- | --- | --- | --- |
| Glabella | G | The most prominent midline point in the midsagittal plane between the eyebrows | Midline |
| Soft tissue nasion | STN | The point of greatest concavity in the midline between the forehead and the nose | Midline |
| Subnasale | Sn | The point at which the columella (nasal septum) merges with the upper lip in the midsagittal plane | Midline |
| Lip peak | Lp(L)/Lp(R) | The highest point of the upper lip | bilateral |
| Upper lip concave | Lu | The most concave point of the mucocutaneous border of the left and right upper lip peak | Midline |
| Labrale superius | Ls | The anterior and lower point of the middle upper lip | Midline |
| Labrale inferior | Li | The most anterior aspect of the lower vermilion border of the lower lip in the centerline | Midline |
| Soft tissue gnathion | STGn | The most anterior and inferior point on the soft tissue chin in the midsagittal plane | Midline |
| Soft tissue menton | STMe | The most inferior point on the soft tissue chin | Midline |
| Soft tissue alare | Sal(L)/Sal(R) | The most lateral point on each alar contour | bilateral |
| Zygion | Zg(L)/Zg(R) | The most protruded point of each zygomatic arch | bilateral |
| Tragus | Tr(L)/Tr(R) | The most lateral and posterior point of the tragus of the ear | bilateral |
| Soft tissue gonion | Go(L)/Go(R) | The posterolateral turning point of the mandible inferior border and ramus | bilateral |
| Cheilion | Ch(L)/Ch(R) | The most lateral point located at each labial commissure | bilateral |
| Soft tissue mental tubercle | Mt(L)/Mt(R) | The inferior turning point of the chin and mandible inferior border | bilateral |
| Soft tissue supramental | STB | The most concave point in the center of mental fossa | Midline |
| Pronasale | Pn | The most prominent midline point on the tip of the nose | Midline |
| Endocanthion | En(L)/En(R) | The point at the inner commissure of the eye fissure | bilateral |
| Exocanthion | Ex(L)/Ex(R) | The point at the outer commissure of the eye fissure | bilateral |
| Highest eyebrow | He(L)/He(R) | The highest point of the eyebrow | bilateral |
| Pupil | P(L)/P® | The point of the pupil | bilateral |

**Supplemental Figures**

Figure S1. Artificial transformation of Ch’s coordinates in the wireframe 3D facial mask, related to Figure 5.

A-C: Illustration of 3D displacement of one Ch point in different views; dark blue arrow: change in Z-dimension (sagittal), green arrow: change in Y-dimension (vertical), light blue arrow: change in X-dimension (transverse). D: Changes in angle parameters during Ch-point transplantation.


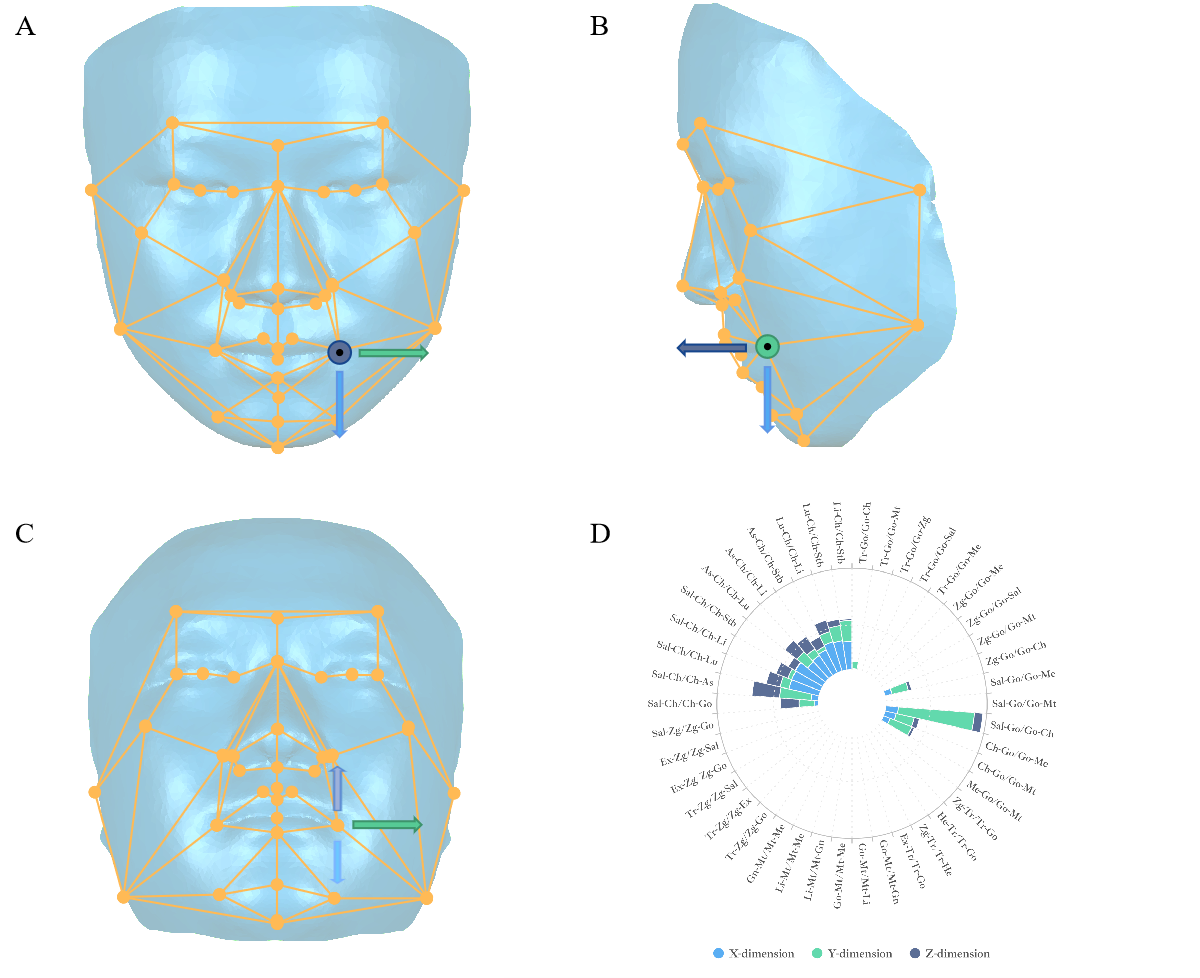


Figure S2. Artificial transformation of Zg’s coordinates in the wireframe 3D facial mask, related to Figure 5.

A-C: Illustration of 3D displacement of one Zg point in different views; dark blue arrow: change in Z-dimension (sagittal), green arrow: change in Y-dimension (vertical), light blue arrow: change in X-dimension (transverse). D: Changes in the angle parameters during Zg point transplantation.


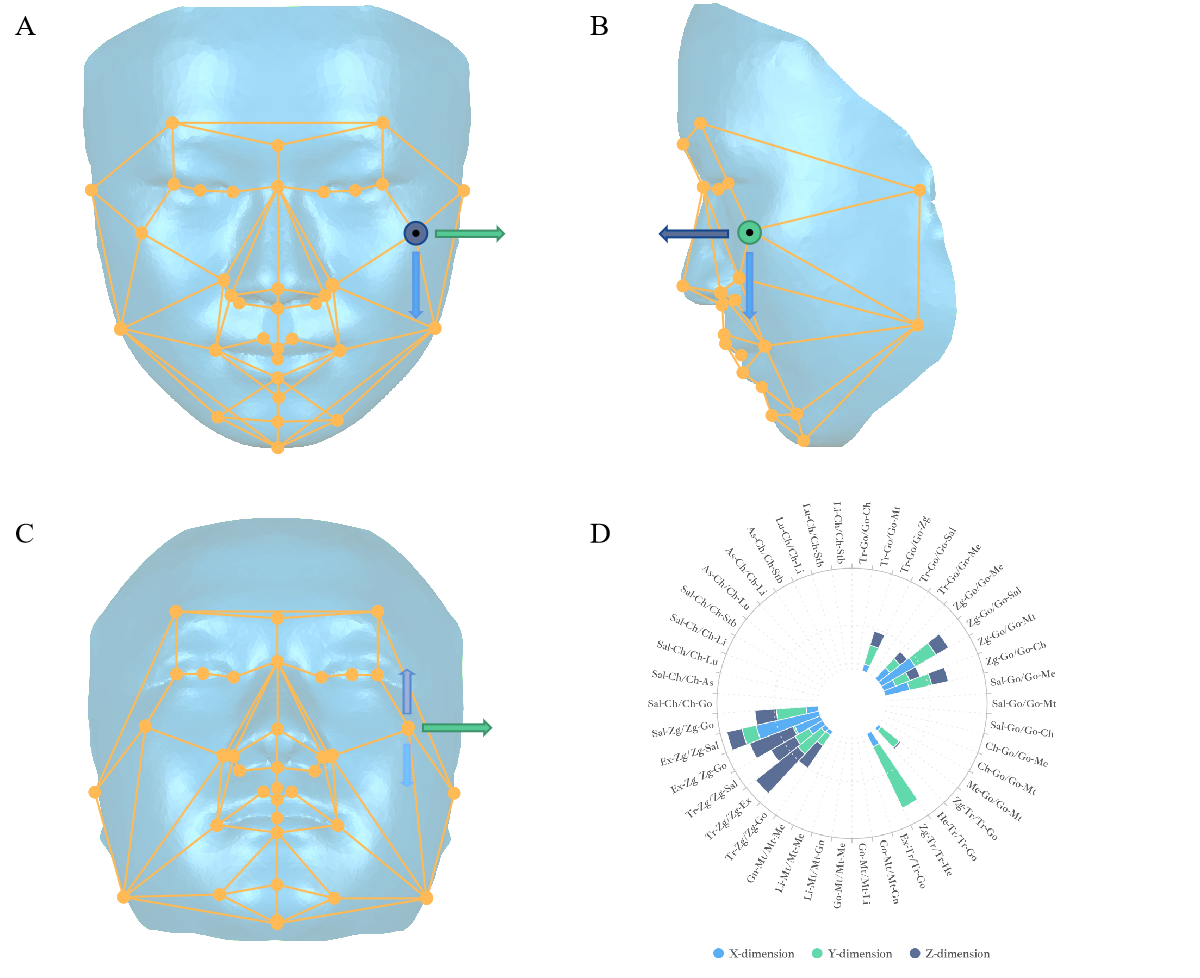


Figure S3. Artificial transformation of Mt’s coordinates in the wireframe 3D facial mask, related to Figure 5.

A-C: Illustration of 3D displacement of one Mt point in different views; dark blue arrow: change in Z-dimension (sagittal), green arrow: change in Y-dimension (vertical), light blue arrow: change in X-dimension (transverse). D: Changes in the angle parameters during Mt point transplantation.


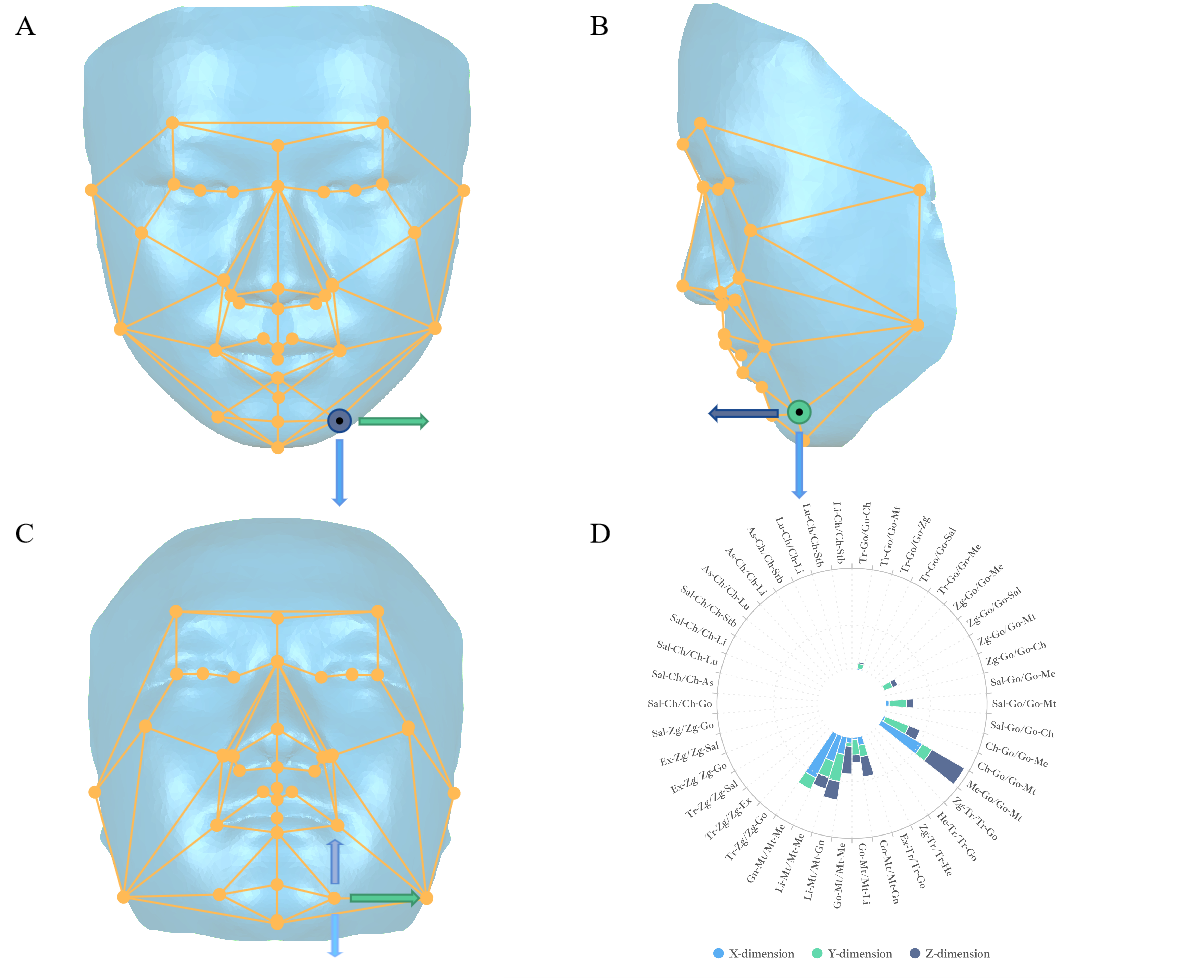


Figure S4. Artificial transformation of Tr’s coordinates in the wireframe 3D facial mask, related to Figure 5.

A-C: Illustration of 3D displacement of one Tr point in different views; dark blue arrow: change in Z-dimension (sagittal), green arrow: change in Y-dimension (vertical), light blue arrow: change in X-dimension (transverse). D: Changes in the angle parameters during Tr point transplantation.


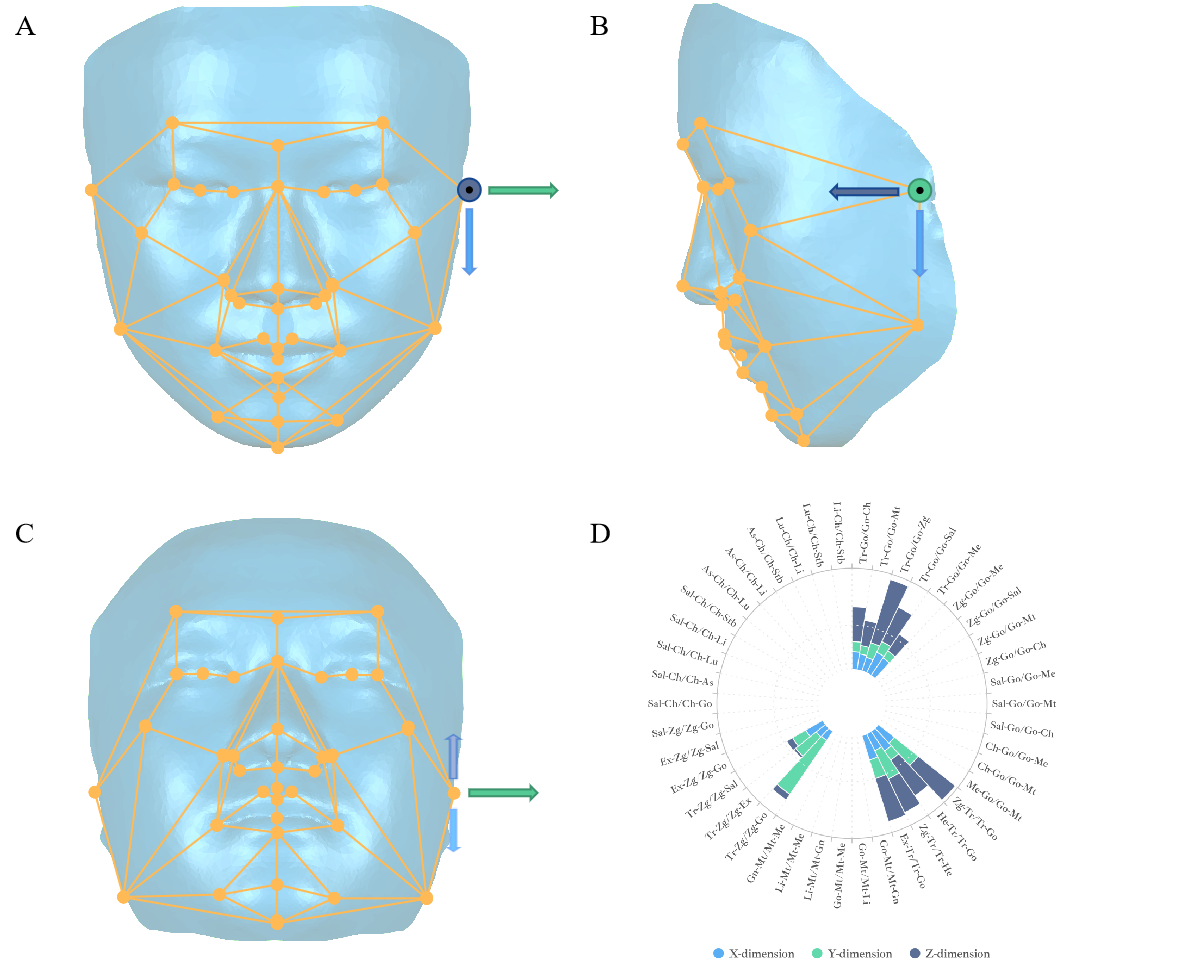


Figure S5. Illustration of the angular parameters selected for five facial parts in 3D


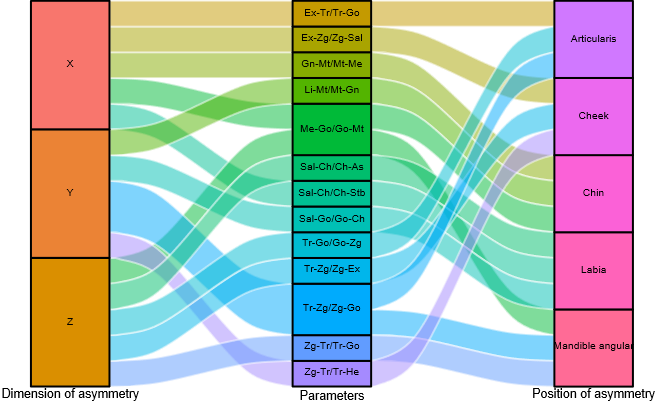

Supplement: Multimedia component 1 [file mmc1.docx]
